# In Vitro Evaluation of Postbiotics Produced from Bacterial Isolates Obtained from Rainbow Trout and Nile Tilapia against the Pathogens *Yersinia ruckeri* and *Aeromonas salmonicida* subsp. *salmonicida*

**DOI:** 10.3390/foods12040861

**Published:** 2023-02-17

**Authors:** Mario Quintanilla-Pineda, Chajira Garrote Achou, Jesús Díaz, Ana Gutiérrez-Falcon, María Bravo, Juan Ignacio Herrera-Muñoz, Nelson Peña-Navarro, Carlos Alvarado, Francisco C. Ibañez, Florencio Marzo

**Affiliations:** 1 Departamento de Fisiología y Nutrición Animal, Universidad Pública de Navarra, 31006 Navarre, Spain; 2 Productos Específicos de Nutrición y Técnicas Alimentarias SL, 31191 Navarre, Spain; 3 Innovación en Gestión y Conservación de Ungulados, 10004 Cáceres, Spain; 4 Escuela de Zootecnia, Universidad de Costa Rica, San José 11501, Costa Rica; 5 Departamento de Acuicultura, Universidad Técnica Nacional, Puntarenas 60115, Costa Rica; 6 Instituto Costarricense de Pesca y Acuicultura, Puntarenas 60101, Costa Rica

**Keywords:** furunculosis, yersiniosis, *Weissella*, biological tools, antibacterial, microbiota, diseases, alternative treatment methods

## Abstract

The use of antibiotics in aquaculture leads to the proliferation of multidrug-resistant bacteria, and an urgent need for developing new alternatives to prevent and control disease has, thus, arisen. In this scenario, postbiotics represent a promising tool to achieve this purpose; thus, in this study, isolation and selection of bacteria to further produce and evaluate their postbiotics antibacterial activity against fish pathogens was executed. In this respect, bacterial isolates from rainbow trout and Nile tilapia were obtained and tested in vitro against *Yersinia ruckeri* and *Aeromonas salmonicida* subsp. *salmonicida*. From 369 obtained isolates, 69 were selected after initial evaluation. Afterwards, additional screening was carried out by spot-on-lawn assay to finally select twelve isolates; four were identified as *Pediococcus acidilactici*, seven as *Weissella cibaria*, and one as *Weissella paramesenteroides* by matrix assisted laser desorption/ionization, time-of-flight mass spectrometry (MALDI-TOF MS). Selected bacteria were used to obtain postbiotic products to test their antagonistic activity through coculture challenge and broth microdilution assays. The influence of incubation time prior to postbiotic production on antagonistic behavior was also recorded. Two isolates identified as *W. cibaria* were able to significantly reduce (*p* < 0.05) *A. salmonicida* subsp. *salmonicida*’s growth in the coculture challenge up to 4.49 ± 0.05 Log CFU/mL, and even though the reduction in *Y. ruckeri* was not as effective, some inhibition on the pathogen’s growth was reported; at the same time, most of the postbiotic products obtained showed more antibacterial activity when obtained from broth cultures incubated for 72 h. Based on the results obtained, the preliminary identification of the isolates that expressed the highest inhibitory activity was confirmed by partial sequencing as *W. cibaria*. Through our study, it can be concluded that postbiotics produced by these strains are useful to inhibit the growth of the pathogens and could, thereby, be applicable in further research to develop suitable tools as feed additives for disease control and prevention in aquaculture.

## 1. Introduction

Modern aquaculture makes it possible to produce large quantities of fish at low economic cost using facilities designed to accommodate high stock biomass of animals, which, among other determinant factors, has increased the occurrence of disease outbreaks in farmed animals [1]. This intensive condition has caused disturbance of the pathogen–host–environment interaction, leading to the use of antibiotic agents in the aquaculture industry to protect fish against diseases [2], and although this approach to disease is common, it has contributed to the selection, persistence, and spread of antibiotic-resistant bacteria, endangering both human and animal health [3].

In aquaculture rearing, pathogenic bacteria have been described in most of the taxonomic groups, with *Aeromonas salmonicida*, the causative agent of furunculosis, and *Yersinia ruckeri*, the causative agent of enteric red mouth disease (ERMD) or yersiniosis, being some of the most important ones that affect rainbow trout farmers [4,5].

At present, alternatives such as vaccines have emerged as an option, although their application has some limitations, such as the fact that they do not offer complete efficacy when applied to juvenile fish, which can lead to intensive labor when injections are required [6], as well as complications such as adhesions and pigmentation of the abdomen, spinal deformation, retarded growth, and autoimmune symptoms [7].

In this scenario, interest has been paid to the beneficial microbes that are normally found in healthy fish [8]. These microorganisms, known as probiotics, have a part in the host defense mechanisms against pathogens as a natural defensive barrier [9]. In rainbow trout, most of the probiotics used for this purpose come from terrestrial sources [2]; nevertheless, bacteria associated with the rearing water or the host tend to perform better, since they are within their original environment [10].

Microbial strains are able to produce antimicrobial compounds [11]; these components, among the remaining microbial cell structures after inactivation, microbial metabolites, and end products of bacterial growth in the absence of viable cells have been named postbiotics, defined as “preparations of inanimate microorganisms and/or their components that confer a health benefit on the host” [12]. They represent a great alternative option when referring to biological approaches for disease control, since their application can be safer and more stable compared to products that require live microorganisms to be functional [13,14].

Studies have proven how beneficial postbiotics can be for the aquaculture industry through in vitro experiments by inhibiting the growth of fish pathogens such as *Photobacterium damselae* subsp. *piscicida*, *Vibrio anguillarum*, and *Lactococcus garvieae* [15,16,17].

Other in vivo studies have focused on changes in gut bacterial community composition as well as protection against the pathogen *L. garvieae* after dietary supplementation with postbiotics in rainbow trout [18]. The benefits of these biological tools have also proved to be effective in promoting growth performance in sturgeon and white shrimp [19,20]. The results obtained by these studies evidence the importance of continuing the research on this matter to reinforce the range of beneficial properties that postbiotics might provide.

At the present, however, there are no findings on the antagonistic effects of postbiotics against *A. salmonicida* subsp. *salmonicida* and *Y. ruckeri*. In the present study, the hypothesis is that postbiotics obtained from bacterial strains isolated from fish natural microbiota with prophylactic and therapeutic activity against pathogens would be suitable for the aquaculture industry. Therefore, the objectives of the work were to isolate morphologically different colonies from rainbow trout and Nile tilapia to evaluate their antibacterial effect against *Y. ruckeri* and *A. salmonicida* subsp. *salmonicida* as live cells. Consequently, isolates that could inhibit the growth of pathogens were inactivated to obtain postbiotics and demonstrate that their inhibitory activity remains after inactivation through coculture and broth microdilution assay challenges. The influence of the incubation time of the selected isolates before inactivation on the antibacterial effect was studied as well as changes in pH and bacterial concentration through time.

## 2. Materials and Methods

### 2.1. Sample Collection

A total of 42 fully grown healthy fish fed with commercial diets were used to isolate bacterial strains. Samples from the gut, gill, and skin mucus were collected according to Salinas et al. [8]. The species included for sampling were: 20 individuals of *Oncorhynchus mykiss* of 388.72 ± 80.45 g from Piscifactoría Leitza SL (Leitza, Spain), 11 individuals of *Oreochromis niloticus* of 245.45 ± 43.71 g from Enrique Jiménez Experimental Station (Cañas, Costa Rica), and 11 individuals of *O. niloticus* of 253.75 ± 80.36 from Los Diamantes Experimental Station (Punto Jimenez, Costa Rica) [8]. Collected samples from Costa Rica were authorized by the Comisión Nacional para la Gestión de la Biodiversidad (CONAGEBIO, San José, Costa Rica) under Project ID 1098. Animals were sacrificed with an overdose of 200 mg/L tricaine methanesulfonate solution Pharmaq Ltd. (MS 222, Crowborough, East Sussex, England) according to the recommendations of the Real Decreto 53/2013 of the consolidated legislation of Spain.

Each sample was taken aseptically using an invasive sterile sample collection swab. Skin mucus was collected from the right lateral part of the fish, gill mucus samples were taken by lifting the operculum and dragging the swab through the filaments. Gut mucus was collected by dissecting the fish to pull out the mid-section of the intestine; the lumen was rinsed with 0.9% sterile saline solution to detach the intestinal content, and then the mucous membrane was exposed by making a longitudinal cut to finally detach the mucus by scraping the internal walls of the organ. Samples were placed into individual sterile Eppendorf of 1.5 mL and stored in ice for transportation. After arrival at the laboratory, each collected sample was suspended in 0.9 mL of buffered peptone water 2% (Biolife, Milan, Italy), and 1.0 mL of glycerol anhydrous 30% Scharlab S.L. (Barcelona, Spain) and preserved at −20 °C until processing.

### 2.2. Isolation of Bacterial Strains

The samples were then fold diluted in 2% buffered peptone water and 0.1 mL of each dilution was spread over the surface of plates of Trypticase Soy Agar Laboratorios Conda S.A. (TSA, Madrid, Spain) and Man Rogosa and Sharpe Agar Laboratorios Conda S.A. (MRS-A, Madrid, Spain) [21]. Inoculated plates were incubated at 22 °C for 48 h in aerobiosis. The selection was made based on morphology, color, and brightness according to Alonso et al. [22]. A maximum of 10 isolates per sample were selected and purified by subsequently streaking on a fresh medium.

Isolate codes were assigned in the following order: the number of sampled fish; letter M, I, or B, stands for isolates obtained from skin, gut, or gill mucus, respectively, and the second letter of the code distinguishes isolates obtained from the same sample.

### 2.3. Pathogens

The pathogenic strains used for this study come from disease outbreaks in rainbow trout farms in Spain and Italy. *Aeromonas salmonicida* subsp. *salmonicida* was isolated in 2019 in Navarre (Spain), and *Yersinia ruckeri* strain was isolated in 2020 in Brescia (Italy). Pathogens were identified by qPCR (real-time quantitative polymerase chain reaction). For further studies, Trypticase Soy Agar and broth Laboratorios Conda S.A. (TSA/TSB, Madrid, Spain) was used for *Yersinia ruckeri* culture and Müller Hilton agar-broth Laboratorios Conda S.A. (MH/MH agar, Madrid, Spain) for *A. salmonicida* subsp. *salmonicida*; for all further experiments, pathogens were incubated in aerobic conditions at 30 °C, preserved at −80 °C in 1.5 mL Eppendorf tubes with 1:1 proportions of glycerol 30% and the corresponding culture broth. Prior to use, the pathogen cryovials were defrosted at 4 °C and manipulated within a laminar flow cabinet.

### 2.4. Inhibitory Activity of Isolates

For the first screening of inhibitory activity, the 369 obtained isolates were cultured in Man Rogosa Sharpe Broth Laboratorios Conda S.A. (MRS-B, Madrid, Spain), to then be challenged against the two pathogens in an alongside culture by the spot-on-lawn assay as described by Bravo et al. [23]. Briefly, a droplet of 2 µL of each isolated was inoculated immediately after seeding the entire surface of a plate of Brain Heart Infusion Agar Liofilchem S.r.l. (BHI-A, Roseto Degli Abruzzi, Italy) with 100 µL of overnight culture of the pathogens and incubated at 30 °C in aerobic conditions for 24 h before reading. The performance of isolates was evaluated by classifying their capacity to grow alongside the pathogens as high (delimited borders between isolates and pathogen growth and/or inhibition zone), intermediate (nondelimited borders between isolate and pathogen growth), and low (absence of isolate growth), and only those who showed height capacity of growing alongside with both pathogens or shown zones of inhibition were chosen for the next set of challenges. The selected isolates were subjected to a new battery of challenges performed in quadruplicate, by rising the volume of isolate culture to 5 µL to promote potential antagonistic activity. Antagonism was measured using the diameter of the inhibition zone around the droplet, and selection was made by tracing their inhibition halos on a normal curve, selecting those isolates that were above the central value for both pathogen challenges, showing significative difference (*p* < 0.05) from 10.00 mm of halo of inhibition.

### 2.5. Preliminary Identification

The identification of selected isolates was made by MALDI-TOF MS using Microflex LT Bruker Daltonics (Berlin, Germany). Processing was made using the software BioTyper 3.4 Bruker Daltonics (Berlin, Germany). The database MBT compass (version 4.1, Bruker Daltonics GmbH & Co. KG, Bremen, Germany) was used for comparing resemblances. The results obtained were interpreted as a lack of identification if the score was <1.7, identification at the gender level if the score was <2.0 and ≥1.7, possible identification at the species level if the core level was <2.3 and ≥2.0, and highly probable at the species level if the score was 3.0 or ≥2.3.

### 2.6. Inactivation Process

Postbiotics were obtained by two different methods, for which aliquots at 24, 48, and 72 h of broth culture from selected isolates were aseptically taken to be processed. The first method was used to obtain cell-free supernatants as described by Shafipour Yordshahi et al. [24] by first exposing the aliquots to heat inactivation at 80 °C for 1 h, to then centrifuge at 4500 rpm for 10 min; the remaining pellets were discarded, and cell-free supernatants were decanted and conserved until use. The second method used for inactivation was executed by heat inactivation at 80 °C for 1 h without centrifugation as described by Choi and Yoon [25] to see if the inanimate remaining suspended bacterial structures change the antagonistic activity of the metabolic products.

To ensure bacterial non-viability of both methods, one single drop of 5 µL of the final product was inoculated on MRS-A and incubated at 30 °C for 48 h; the absence of growth was considered as complete inactivation of the bacterial culture. At each point of sampling and inactivation, a sample from the broth culture was microdiluted using PBS Fisher BioReagents (Geel, Belgium) and inoculated in MRS-A for colony-forming unit counting; the remaining volume was used to measure the pH (PHS-3BW microprocessor pH/MV/Temperature meter, BANTE instrument, Qingdao, China).

### 2.7. Broth Microdilution Assay

The antibacterial activity of cell-free supernatants against both pathogens was evaluated based on Bravo et al. method [26], where the result is measured as the absence of turbidity in broth media inoculated with a known concentration of pathogen and serial dilutions of postbiotics. Briefly, a sterile 96-well microplate was used to make serial dilutions of cell-free supernatants in Brain Heart Infusion Broth Liofilchem S.r.l. (BHI-B, Roseto degli Abruzzi, Italy). Immediately after the dilutions were made, overnight cultures of *Y. ruckeri* and *A. salmonicida* subsp. *salmonicida* were added to the plates. As a positive and negative control, supernatants and pathogens alone with BHI-B were incubated into the same plates. After the microplates were completely assembled, they were covered and incubated at 30 °C for 24 h in aerobic conditions before reading.The results were measured in activity units per milliliter of inhibition activity (AU/mL) defined as the reciprocal of the higher dilution, which shows clear inhibition of pathogen growth expressed as the absence of turbidity in the well [27].

### 2.8. Coculture Challenge

To evaluate the antibacterial activity of the postbiotic product containing inanimate cell structures and metabolites obtained by heat inactivation, challenges were carried out as described by Bravo et al. [26] by inoculating 10 mL of sterile BHI-B supplemented with 1000 µL of heat-inactivated postbiotics to then add 10 µL of an overnight culture of each pathogen, for a final challenge of 1:100 proportion, then inoculated cultures where incubated for 24 h at 30 °C in aerobic conditions. Controls were executed by challenging the pathogens against sterile MRS-B in the same proportions. Once the incubation time had passed, samples of each challenge were taken and microdiluted for colony-forming unit counting. Inhibition was expressed in a reduction in Log of the respective pathogen counting calculated using three biological replicates and then normalized with the number of CFU/mL of *A. salmonicida* subsp. *salmonicida* and *Y. ruckeri* monocultures.

### 2.9. Partial Sequencing of Gene 16s

Molecular identification was made by the Spanish Collection of Type Cultures (CECT, Valencia, Spain) and performed as described by Arahal et al. [28]. Briefly, an active culture of the selected isolates was used for DNA extraction using a Bacterial DNA preparation kit Jena Bioscience (Jena, Germany) following the manufacturer’s instructions, afterward, amplification by PCR was performed in a thermocycler using universal primers, 616V (5′-AGA GTT TGA TYM TGG CTC AG -3′) and 699R (5′-RGG GTT GCG CTC GTT -3′). PCR products were checked by electrophoresis on agarose gel 1 % (*w*/*v*) in Tris-Borate-EDTA buffer at 135 V for 25 min. Fragments of PCR were sequenced using ABI 3730xl sequencer Applied Biosystems (Foster City, California, USA) with the same primers of the PCR at a 5 nM concentration. The 16S rRNA gene sequence was then analyzed on EzTaxon (http://eztaxon-e.ezbiocloud.net/ accessed on 22 May 2022) and BLAST (https://blast.ncbi.nlm.nih.gov/Blast.cgi accessed on 22 May 2022) platforms. The results presented include the percentage of similarity and the accession number of the sequence of the type of strain of the species with the closest identity.

### 2.10. Statistical Analysis

This study was executed following a factorial design, where the isolates, time, and pathogens functioned as independent variables while the colony forming unit counts, pH, inhibition zones (mm), growth reduction counts, and arbitrary units of inhibition functioned as dependent variables. The results are expressed as mean ± standard error (SE). One-way analysis of variance (ANOVA) was used for analysis followed by least significant difference test (LSD) in case the values presented significant differences. Statistically significant difference was set at a level of 0.05. The statistical analyses were performed using InfoStat version 2017 InfoStat group (Cordoba, Argentina).

## 3. Results

### 3.1. Obtained Isolates and Inhibitory Activity against Pathogens

Initially, 369 morphologically distinct bacterial isolates from 149 samples of gut, skin, and gill mucus of rainbow trout and Nile tilapia were collected and archived in cryo-vials at −80 °C (Table 1).

Using the spot-on-lawn assay, from the 369 strains isolated, 69 showed an inhibition zone around the inoculation site on plates seeded with the pathogenic strains *Y. ruckeri* and *A. salmonicida* subsp. *salmonicida*, classifying their antagonistic activity as high in challenges against both pathogens; the final selection was carried out as described in Table 1.

The 69 selected isolates were challenged again, adjusting the volume of isolates to better express the previously recorded antagonistic activity, and only those isolates that had a mean inhibition value superior to the mean value of all challenges made against *A. salmonicida* subsp. *salmonicida* and *Y. ruckeri* were selected for further studies. The results obtained are presented in Table 2. Briefly, the results against *A. salmonicida* subsp. *salmonicida* indicated that the mean value of the diameter of the inhibition halos was significantly higher than 10.00 mm (*p* < 0.05) for all selected isolates, and all the isolates, except for 24IC, 35IH, and 26MA, were significantly higher than 15.00 mm (*p* < 0.05); most of the isolates showed similar behavior, except for the isolates 2MB, 6MC, and 24IC, which had significant differences (*p* < 0.05) from the rest of the isolates results; the first two being the ones who showed greatest inhibition values, and the last one had the lowest inhibition value when compared to the rest of the selected isolates.

On *Y. ruckeri* challenges, halos from all selected isolates (Table 2) were significantly higher than 10.00 mm (*p* < 0.05) and the greatest inhibition values correspond to the isolates 11MH, 10BE, 17MD, 18ME, and 7MB, whose halo diameters were significantly higher than 15.00 mm (*p* < 0.05). Most of the selected isolates showed significantly different (*p* < 0.05) behavior against *Y. ruckeri*, being the only ones who expressed no significative difference (*p* < 0.05) from each other the isolate 35IH with 16BB, 13ID, and 18IC.

### 3.2. Preliminary Identification

Based on the results obtained by MALDI-TOF (Table 3), the isolates 13ID, 17MD, 2MB, 6MC, 9MC, 25MA, and 26MA were scored as highly probable at the species level to the strain *Weissella cibaria* DMS 15878T. The isolate 16BB was scored as highly probable at the species level to the strain *Pediococcus acidilactici* 146 RTL, and the isolates 10BE, 24IC, and 35IB were classified with possible identification at the species level as *Pediococcus acidilactici* 146 RTL; only the isolate 5BB was identified at the gender level with resemblance to *Weissella paramesenteroides* DMS 20288T. Finally, the isolates 7MB, 11MH, 18ME, 18IC, and 35IH scored with possible identification at the species level as *Lactococcus garvieae* 20684T, a strain known to be the causative agent of lactococcosis in rainbow trout; for this reason, they were not considered for the rest of this study.

### 3.3. Postbiotic Production

All isolates showed complete inactivation when exposed to heat at 80 °C for 1 h with or without centrifugation. Changes in pH and Log of CFU/mL at 24, 48, and 72 h of culture were registered (Table 4); most of the isolates showed significative differences (*p* < 0.05) through each point of sampling; particularly, the isolates preliminarily identified as *W. cibaria* show a progressive decline in bacterial concentration, and the isolates W.c.6MC, W.c.9MC, W.c.26MA, W.c.17MD, and W.c.13ID reported a complete loss of viability at 72 h of incubation. The isolate W.c.25MA reported no significative difference (*p* < 0.05) in bacterial concentration at 24 and 48 h, but the reading at 72 h showed significative (*p* < 0.05) lower concentration when compared to the first two points of reading; this isolate did not lose complete viability as seen in other *W. cibaria* behavior. Contrastingly, the isolates P.a.24IC, P.a.35IB, P.a.10BE, and P.a.16BB showed a clear tendency to increase their bacterial concentration trough time, reaching higher concentrations with a significant difference at 48 or 72 h of culture. All isolates showed a significative difference (*p* < 0.05) in the pH of the culture media when compared to the sterile MRS–B, whose pH was 6.40.

### 3.4. Broth Microdilution Assay

**Figure 1:**
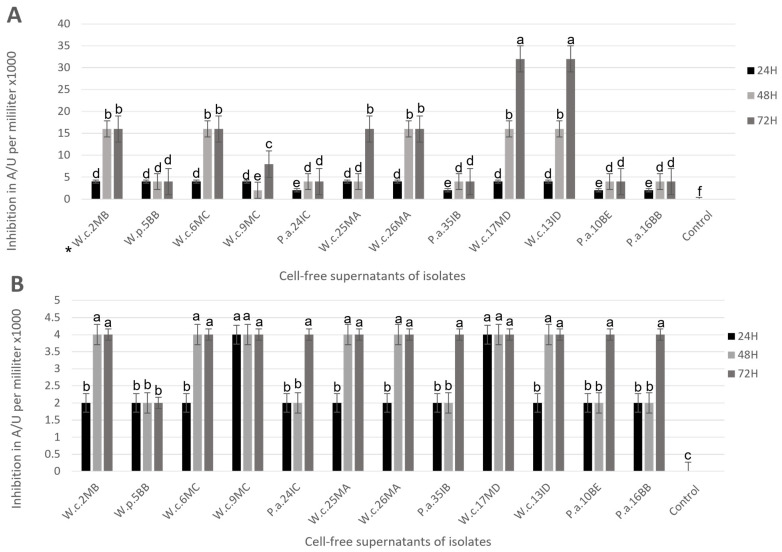
Microdilution broth assay of cell-free supernatants produced from 12 isolates at three points at 72 h of incubation before inactivation. (**A**) Challenges against *A. salmonicida* subsp. *salmonicida.* (**B**) Challenges against *Y. ruckeri.* Error bars express SE (*n* = 3). Bars with different superscripts are significantly different (*p* < 0.05). Results are expressed as arbitrary units of inhibitory activity per milliliter AU/mL defined as the reciprocal of the greatest dilution showing clear inhibition of the pathogen. * For codifications, refer to Table 2 and Table 3.

As shown in Figure 1, all cell-free supernatants obtained from heat inactivation and centrifugation show certain inhibition against both pathogens. In *A. salmonicida* subsp. *salmonicida* challenges (Figure 1A), the products obtained from isolates P.a.10BE, P.a.16BB, P.a.24IC, and P.a.35IB reached 2000 AU/mL of inhibitory activity at 24 h and 4000 AU/mL at 48 and 72 h. On the other hand, postbiotics obtained from isolates W.c.2MB, W.c.6MC, and W.c.26MA reached their higher potential at 48 h, when their inhibitory activity increased up to 16,000 AU/mL when compared to the product obtained at 24 h. Contrastingly, the isolates W.c.9MC and W.c.25MA reached their higher potential at 72 h with 16,000 UA/mL of inhibition. However, the isolates W.c.13ID and W.c.17MD showed a peak of inhibitory activity at 72 h that increased to 32,000 AU/mL of inhibition, the highest inhibitory activity expressed in all challenges. The postbiotics obtained from isolate W.p.5BB showed the lowest results in this set of experiments with 2000 AU/mL of inhibitory activity.

In *Y. ruckeri* challenges (Figure 1B), postbiotics obtained from isolates P.a.10BE, P.a.16BB, P.a.24IC, and P.a.35IB reached 2000 AU/mL of inhibitory activity at 24 and 48 h, and their inhibitory activity increased to 4000 AU/mL when cell-free supernatants were obtained after 72 h of incubation. Postbiotics obtained from the isolates W.c.13ID, W.c.2MB, W.c.6MC, W.c.25MA, and W.c.26MA showed an increase in inhibitory activity up to 4000 AU/mL at 48 h when compared to 24 h, when they showed 2000 AU/mL of inhibitory activity; their inhibitory activity continues to be the same as 48 h at 72 h. The postbiotics obtained from the isolates W.c.17MD and W.c.9MC reached 4000 AU/mL of inhibitory activity at 24 h and remains the same at 48 and 72 h. Last, isolate W.p.5BB showed 2000 AU/mL of inhibition activity in the entire set of challenges.

### 3.5. Coculture Challenge

In the coculture challenge, changes in the antagonistic activity of postbiotics produced by heat inactivation with suspended bacterial structures at 24, 48, and 72 h of incubation using 12 isolates were evaluated against the pathogens *Y. ruckeri* and *A. salmonicida* subsp. *salmonicida* (Table 5). The reduction in pathogen growth was calculated using three biological replicates and then normalized with the number of CFU/mL of the respective pathogen.

Results of the coculture challenge indicated that the isolates W.c.2MB, W.c.6MC, W.c.9MC, W.c.25MA, W.c.26MA, W.c.17MD, and W.c.13ID had significatively (*p* < 0.05) higher antimicrobial activity against *A. salmonicida* subsp. *salmonicida* at 48 and 72 h of incubation before inactivation; the isolates W.c.17MD and W.c.13ID showed the greatest value of inhibition when compared to the rest of the treatments at 72 h of culture before inactivation, as seen in the broth microdilution assay. No significative reduction (*p* < 0.05) in *A. salmonicida* subsp. *salmonicida* growth was observed when treated with postbiotics produced at 24 h. Significative reduction (*p <* 0.05) in *Y. ruckeri* growth was reported at 24, 48, and 72 h; nevertheless, the reduction in Log of CFU/mL was not as effective as the one observed against *A. salmonicida* subsp. *salmonicida*, being inferior to 1 Log CFU/mL reduction. Either way, the results obtained at 72 h in *Y. ruckeri* challenges showed the highest reductions when compared to those observed at 48 and 72 h, which matches with the antibacterial activity behavior observed in *A. salmonicida* subsp. *salmonicida* challenges.

**Figure 2:**
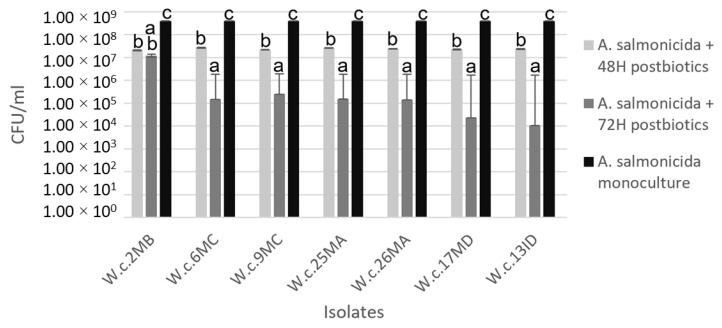
Comparison of growth expressed in coculture challenge of *A. salmonicida* subsp. *salmonicida* treated with postbiotics obtained from 7 isolates preliminarily identified as *W. cibaria* incubated for 48 and 72 h before inactivation. Error bars express SE (*n* = 3). Bars with different superscripts are significantly different (*p* < 0.05).

Reduction in CFU/mL of *A. salmonicida* subsp. *salmonicida* when cultured with postbiotics produced by the isolates W.c.2MB, W.c.6MC, W.c.9MC, W.c.25MA, W.c.26MA, W.c.17MD, and W.c.13ID is shown in Figure 2; particular interest should be paid to the results obtained from the isolates W.c.17MD and W.c.13ID at 72 h, where CFU/mL of *A. salmonicida* subsp. *salmonicida* was reduced by four exponentials when compared to the control.

### 3.6. Partial Sequencing of Gene 16s

Based on the results obtained from the broth microdilution assay and coculture challenge, the isolates W.c.13ID and W.c.17MD were selected for partial sequencing identification of the 16s rRNA gene. The results showed 100% of similarity *to W. cibaria* (974/974 bp) for the isolate W.c.17MD, and 100% of similarity to *W. cibaria* (1007/1007 bp). The type of strain used to compare resemblance from obtained results was *W. cibaria* AEKT01000037 (KACC11862^T^). Results obtained from partial sequencing of the gene 16s confirm the accuracy of the results obtained on preliminary identification by MALDI-TOF.

## 4. Discussion

*Weissella cibaria* is a Gram-positive bacterium with short rods that grow in pairs, forming greyish-white colonies when grown in agar. This genus was originally classified as a *Lactobacillus*, but after deeper research of its genome, it was renamed, now known as *Weissella* [29]. It has been studied for its metabolites and their capacity to stimulate other probiotic bacteria growth [30], as well as the inhibition of unwanted bacteria on fermented products and periodontal pathogens [31,32]. *W. cibaria* has been isolated from food, environmental, and animal sources, including the fish species rainbow trout, and crucian carp [33].

Studies such as ours have been carried out, where the antagonism of different strains of the *Weissella* genus has been confirmed against several fish pathogens such as *Y. ruckeri*, *Streptococcus agalactiae*, *Photobacterium piscicida*, *Aeromonas hydrophila*, *Vibrio anguillarum*, and *Edwardsiella tarda* [34,35]. Nevertheless, no in vitro or in vivo studies to control or prevent *A. salmonicida* subsp. *salmonicida* and *Y. ruckeri* using *W. cibaria* or *W. paramesenteroides* have been found. Furthermore, studies have confirmed the beneficial effect of supplementing fish feed with *W. cibaria* as a probiotic, improving non-specific immune parameters and increasing survival rates after *Aeromonas veronii* infection in crucian carp [36], as well as increasing red blood concentration in South American catfish, improving the oxygen supply to fish tissues [37]. Despite its beneficial effect as a probiotic having been proven, no studies on its postbiotic products and their application for the aquaculture industry have yet been recorded.

On the other hand, the species *P. acidilactici* has been widely studied as a probiotic strain in aquaculture as a feed additive, improving the peripheral and intestinal immunity of Nile tilapia and reducing mortalities in white shrimp under *Vibrio* spp. infection [38,39]. However, in our study, the strains identified as *P. acidilactici* did not show significative antagonistic activity for challenges made against both pathogens on the two executed techniques after cell inactivation.

Even though the usefulness of *W. cibaria* has been pointed out for its capacity of producing non-digestible oligosaccharides and extracellular polysaccharides in fermented food, feed, clinical, and cosmetics industries [40], its applications as a postbiotic in the aquaculture industry, as well as the changes in its antagonistic activity under different incubation conditions, have not been recorded. In that respect, our study was focused on the changes in expression in antibacterial activity of the obtained isolates at different times of incubation, as well as their changes in bacterial counts and pH at 72 h of incubation, in order to establish the right formula for creating a novel feed additive that can be delivered to early-life-stage fish, produce a product that will remain stable for longer periods of time since no viable microorganisms would be part of the final product, and become an economically viable option that can be managed under less strict conditions than products that require cold chains to stay viable or high technology to be synthesized.

For which the fish species rainbow trout (*O. mykiss*) and Nile tilapia (*O. niloticus*) were used for the isolation of bacteria, the specimens selected for the study were full-grown and healthy animals, and the organs used for the collection of mucus samples the were gut, gill, and skin. To make the right selection of bacteria whose postbiotics were going to be studied for their antagonistic activity against *A. salmonicida* subsp. *salmonicida* and *Y. ruckeri*, the selected isolates must have had antagonistic activity as live cells; hence, from 369 isolations made (Table 1), 69 of them were selected to run a preliminary screening of antagonistic activity by spot-on-lawn assay (Table 1); the isolates W.c.2MB, W.p.5BB, W.c.6MC, W.c.9MC, P.a.24IC, W.c.25MA, P.a.35IB, W.c.17MD, W.c.13ID, P.a.10BE, and P.a.16BB were selected since they showed a significatively higher (*p* < 0.05) halo of inhibition than 10.00 mm, some of them reaching a significatively higher (*p* < 0.05) halo of inhibition than 15.00 mm (Table 2) against both pathogens. MALDI-TOF MS was used as a preliminary identification method since it was considered to provide fast, low-cost, and reliable results [41]; this identification allowed the exclusion of the preliminarily selected strains identified as *L. garvieae*.

To establish whether postbiotics produced from the 12 selected isolates preserve their inhibitory activity against *Y. ruckeri* and *A. salmonicida* subsp. *salmonicida*, the inactivated product obtained by each selected isolate was tested, as was the variation in their antibacterial activity when obtained at different hours of incubation before inactivation since it has been demonstrated that changes in antagonism can be found at different hours of incubation [15]. The changes observed in the current study can be attributed to differences in the concentration of the responsible metabolites of inhibition of pathogen growth (Table 4 and Table 5). The results obtained in the broth microdilution assay (Figure 1) showed inhibition of growth of both pathogens, nevertheless, challenges made against *Y. ruckeri* were less effective than those made against *A. salmonicida* subsp. *salmonicida,* where the isolates W.c.17MD and W.c.13ID reached 32,000 AU/mL of inhibition at 72 h of incubation before inactivation. In the coculture challenges, all isolates preliminarily identified as *W. cibaria* showed substantial inhibition of *A. salmonicida* subsp. *salmonicida* at 48 h, but the greatest reduction in the pathogen growth was recorded at 72 h of incubation before inactivation.

Emphasis should be placed on isolates W.c.17MD and W.c.13ID, who recorded the highest antimicrobial activity in the coculture challenge when compared to the rest of the isolates at 72 h of incubation (Table 5), reducing a total of 4.09 and 4.49, respectively, of the Log concentration of *A. salmonicida* subsp. *salmonicida.* This is ideal for subsequent studies. The results obtained from isolates preliminarily identified as *P. acidilactici* showed less antagonistic activity for challenges made against both pathogens on the two executed techniques. The final selection of the strain was made based on the challenges performed with the pathogenic strain *A. salmonicida* subsp. *salmonicida*, since postbiotic products showed greater antibacterial activity against it, and even though a significative difference (*p* < 0.05) was observed for *Y. ruckeri* growth inhibition, differences among postbiotics were less revealing.

Other studies have demonstrated that the inhibition of pathogens with the preparation of inactivated microorganisms might be mediated by extracellular metabolites such as lipopeptides, surfactins, bacteriocins, and bacteriocin-like inhibitors secreted to the culture medium after bacterial lysis or through bacterial metabolism [2]. Regarding this matter, *W. cibaria* has been confirmed to be a producer of organic acids, water-soluble polymers, and hydrogen peroxide, substances that can inhibit other bacterial growth [32,37]. Other in silico studies have demonstrated that *W. cibaria* has clusters of precursor genes to produce antimicrobial substances such as ethanol and lactate, as well as genes associated with the immunomodulation of the host immune system [42]. Nevertheless, in our study, even though the specific products responsible for the antagonistic activity remain unknown at this level of the investigation, the theoretical presence of some of them could explain the results obtained by all isolates preliminarily identified as *W. cibaria* in the broth microdilution assay and coculture challenge against *A. salmonicida* subsp. *salmonicida* (Figure 1 and Table 5), particularly the ones observed for the strains W.c.17MD and W.c.13ID. At the same time, it has also been demonstrated that the substances responsible for the antagonistic activity that inhibit the growth of the pathogens do not lose effectivity after exposing the broth culture to heat and centrifugation to inactivate the live cells, making it more easily applicable at the industrial level for feed additive purposes.

In conclusion, the results obtained in this study have revealed that the isolated strains belonging to the genus *Weissella* have great potential to inhibit the growth of the pathogen *A. salmonicida* subsp. *salmonicida*, and although the antagonism against *Y. ruckeri* was not as effective, a reduction in the pathogen growth was reported. Through this research, it has been proven that the time of incubation before inactivation does influence antagonistic behavior, as well as the fact that *W. cibaria’s* most valuable results are obtained when the bacterial concentration naturally reduces to zero; in addition, the antibacterial behavior of cell-free supernatants and the product containing bacterial structures and metabolites showed similar results in terms of inhibition increase through time, which might lead to the conclusion that the products responsible for the reported antimicrobial activity are soluble in the culture media and are independent of the lysed bacterial structures suspended in it. Further investigation must be made to identify the responsible compounds of the antibacterial activity expressed by the two postbiotic products obtained from the selected isolates, as well as an assessment of the safety of the postbiotic preparation in aquaculture species and stability of the product under fish digestive tract conditions, among other benefits that these products might bring as a feed additive for the aquaculture industry.

## Figures and Tables

**Table 1:** Number of isolates obtained and selected from three collection sites.

Species	n	Location	Organ					
			Gut		Gill		Skin	
			Obtained	Selected	Obtained	Selected	Obtained	Selected
*O. mykiss*	20	43°04′38.4″ N 1°55′08.9″ W	43	11	97	3	95	11
*O. niloticus*	11	10°20′51.5″ N 85°08′13.8″ W	31	9	11	4	18	6
	11	10°12′41.6″ N 83°46′21.9″ W	30	7	13	7	31	11
Total	42	3	104	27	121	14	144	28

**Table 2:** Inhibition zones (mm) against *Y. ruckeri* and *A. salmonicida* subsp. *salmonicida* produced by selected bacteria, using spot-on-lawn assay.

Isolate *	*Y. ruckeri*	*A. salmonicida* subsp. *salmonicida*
7MB	17.68 ± 0.28 ^a^	18.97 ± 0.66 ^c^
10BE	16.48 ± 0.54 ^ab^	17.46 ± 0.84 ^efg^
11MH	16.75 ± 0.71 ^ab^	17.16 ± 0.68 ^def^
13ID	16.03 ± 0.65 ^bc^	21.51 ± 0.97 ^b^
16BB	16.37 ± 0.77 ^bc^	18.13 ± 1.01 ^de^
17MD	17.17 ± 0.61 ^a^	17.26 ± 0.79 ^efg^
18ME	17.19 ± 0.89 ^a^	17.75 ± 1.03 ^de^
18IC	16.05 ± 0.84 ^bc^	17.68 ± 0.72 ^defg^
2MB	13.61 ± 0.21 ^fg^	23.45 ± 0.90 ^a^
5BB	13.67 ± 0.70 ^fg^	21.36 ± 0.06 ^b^
6MC	12.79 ± 0.91 ^g^	24.45 ± 0.31 ^a^
9MC	13.09 ± 0.30 ^g^	18.18 ± 0.21 ^cde^
24IC	14.33 ± 0.82 ^ef^	15.76 ± 0.67 ^h^
25MA	14.45 ± 0.48 ^ef^	18.80 ± 0.40 ^cd^
26MA	13.78 ± 0.34 ^fg^	16.73 ± 1.10 ^fgh^
35IB	15.05 ± 0.42 ^de^	19.05 ± 0.91 ^c^
35IH	14.48 ± 0.44 ^cd^	16.17 ± 0.38 ^gh^

Results are expressed as means ± SD (*n* = 4) means in the same column with different superscripts are significantly different (*p* < 0.05), * Isolates are codified according to: number means the number of sampled fish; letter stands for: M isolates obtained from skin mucus, B isolates obtained from gill mucus, and I isolates obtained from gut mucus; the second letter of the code distinguishes isolates made from the same sample.

**Table 3:** Preliminary identification of selected isolates by MALDI-TOF.

Isolate Code *	Bacterial Identification	Log Score	Database Resemblance
7MB	*Lactococcus garvieae*	2.16	20684T
P.a.10BE	*Pediococcus acidilactici*	2.08	146 RTL
11MH	*Lactococcus garvieae*	2.20	20684T
W.c.13ID	*Weissella cibaria*	2.37	DSM 15878T
P.a.16BB	*Pediococcus acidilactici*	2.38	146 RTL
W.c.17MD	*Weissella cibaria*	2.41	DSM 15878T
18ME	*Lactococcus garvieae*	2.19	20684T
18IC	*Lactococcus garvieae*	2.13	20684T
W.c.2MB	*Weissella cibaria*	2.34	DSM 15878T
W.c.5BB	*Weissella paramesenteroides*	1.96	DSM 20288T
W.c.6MC	*Weissella cibaria*	2.31	DSM 15878T
W.c.9MC	*Weissella cibaria*	2.35	DSM 15878T
P.a.24IC	*Pediococcus acidilactici*	2.01	146 RTL
W.c.25MA	*Weissella cibaria*	2.32	DSM 15878T
W.c.26MA	*Weissella cibaria*	2.37	DSM 15878T
P.a.35IB	*Pediococcus acidilactici*	2.14	146 RTL
35IH	*Lactococcus garvieae*	2.28	20684T

* The letter added to the front of the code indicates the species of the isolate to better understand further analyses. W.c. stands for *W. cibaria*, P.a. stands for *P. acidilactici*, and W.p. stands for *W. paramesenteroides*.

**Table 4:** Changes in counts (expressed as Log CFU/mL) and pH of selected isolates at three points on 72 h of incubation in MRS-B.

Isolate *	24 h		48 h		72 h		MRS-B
	Counts	pH	Counts	pH	Counts	pH	pH
W.c.2MB	9.31 ± 0.02 ^a^	4.42 ± 0.01 ^a^	9.19 ± 0.03 ^b^	4.44 ± 0.01 ^a^	6.84 ± 0.03 ^c^	4.44 ± 0.01 ^a^	6.40 ± 0.00
W.p.5BB	9.33 ± 0.01 ^a^	4.41 ± 0.00 ^a^	8.69 ± 0.09 ^b^	4.43 ± 0.00 ^b^	7.42 ± 0.01 ^c^	4.41 ± 0.00 ^a^	6.40 ± 0.00
W.c.6MC	9.45 ± 0.02 ^a^	4.41 ± 0.04 ^a^	9.17 ± 0.03 ^b^	4.41 ± 0.02 ^a^	0.00 ± 0.00 ^c^	4.47 ± 0.00 ^b^	6.40 ± 0.00
W.c.9MC	9.30 ± 0.02 ^a^	4.44 ± 0.03 ^a^	8.93 ± 0.03 ^b^	4.43 ± 0.00 ^a^	0.00 ± 0.00 ^c^	4.46 ± 0.00 ^a^	6.40 ± 0.00
P.a.24IC	9.34 ± 0.02 ^c^	4.62 ± 0.02 ^c^	9.49 ± 0.01 ^b^	4.24 ± 0.00 ^b^	9.57 ± 0.01 ^a^	4.07 ± 0.01 ^a^	6.40 ± 0.00
W.c.25MA	9.31 ± 0.01 ^a^	4.45 ± 0.02 ^a^	9.29 ± 0.02 ^a^	4.48 ± 0.02 ^b^	4.00 ± 0.04 ^b^	4.49 ± 0.00 ^b^	6.40 ± 0.00
W.c.26MA	9.50 ± 0.01 ^a^	4.45 ± 0.03 ^a^	9.19 ± 0.01 ^b^	4.42 ± 0.00 ^ab^	0.00 ± 0.00 ^c^	4.46 ± 0.00 ^b^	6.40 ± 0.00
P.a.35IB	9.08 ± 0.01 ^c^	4.58 ± 0.01 ^c^	9.45 ± 0.01 ^a^	4.22 ± 0.00 ^b^	9.41 ± 0.01 ^b^	4.05 ± 0.01 ^a^	6.40 ± 0.00
W.c.17MD	9.42 ± 0.02 ^a^	4.44 ± 0.01 ^b^	9.24 ± 0.02 ^b^	4.43 ± 0.00 ^a^	0.00 ± 0.00 ^c^	4.49 ± 0.00 ^c^	6.40 ± 0.00
W.c.13ID	9.32 ± 0.01 ^a^	4.42 ± 0.01 ^a^	9.07 ± 0.02 ^b^	4.42 ± 0.00 ^a^	0.00 ± 0.00 ^c^	4.47 ± 0.06 ^a^	6.40 ± 0.00
P.a.10BE	9.32 ± 0.01 ^b^	4.57 ± 0.03 ^c^	9.38 ± 0.01 ^b^	4.24 ± 0.02 ^b^	9.45 ± 0.01 ^a^	4.08 ± 0.00 ^a^	6.40 ± 0.00
P.a.16BB	9.47 ± 0.02 ^b^	4.61 ± 0.02 ^c^	9.53 ± 0.01 ^a^	4.20 ± 0.01 ^b^	9.4 ± 0.01 ^a^	4.05 ± 0.03 ^a^	6.40 ± 0.00

Results are expressed as means ± SD (*n* = 3). Means in the same row with different superscripts are significantly different (*p* < 0.05). All pH reads showed significant difference (*p* < 0.05) from the control, means in the same row with different subscripts are significantly different (*p* < 0.05); * For codifications, refer to Table 2 and Table 3.

**Table 5:** Growth reduction counts (expressed as Log CFU/mL) of *A. salmonicida* subsp. *salmonicida* and *Y. ruckeri* in coculture challenge against postbiotics of 12 isolates extracted at three points in 72 h of incubation.

	*A. salmonicida* subsp. *salmonicida*			*Y. ruckeri*		
Isolate	24 h	48 h	72 h	24 h	48 h	72 h
W.c.2MB	0.13 ± 0.13 ^a^	1.14 ± 0.02 ^a^	1.94 ± 1.02 ^c^	0.18 ± 0.09 ^a^	0.18 ± 0.04 ^a^	0.38 ± 0.10 ^a^
W.p.5BB	0.02 ± 0.07 ^abc^	0.02 ± 0.07 ^cd^	−0.17 ± 0.04 ^d^	0.02 ± 0.07 ^cde^	0.19 ± 0.04 ^a^	0.39 ± 0.13 ^a^
W.c.6MC	−0.01 ± 0.08 ^abc^	1.03 ± 0.06 ^b^	3.32 ± 0.17 ^b^	0.03 ± 0.00 ^bcde^	0.19 ± 0.08 ^a^	0.24 ± 0.11 ^a^
W.c.9MC	0.00 ± 0.09 ^abc^	1.11 ± 0.03 ^ab^	3.07 ± 0.10 ^b^	0.11 ± 0.04 ^abc^	0.25 ± 0.07 a	0.37 ± 0.07 ^a^
P.a.24IC	−0.06 ± 0.05 ^c^	0.00 ± 0.04 ^de^	0.19 ± 0.06 ^d^	0.07 ± 0.05 ^bcd^	0.14 ± 0.04 ^ab^	0.39 ± 0.06 ^a^
W.c.25MA	−0.03 ± 0.10 ^bc^	1.04 ± 0.03 ^b^	3.27 ± 0.07 ^b^	0.04 ± 0.02 ^bcde^	0.24 ± 0.10 ^a^	0.29 ± 0.13 ^a^
W.c.26MA	0.04 ± 0.09 ^abc^	1.08 ± 0.06 ^ab^	3.34 ± 0.16 ^b^	0.08 ± 0.02 ^abcd^	0.15 ± 0.10 ^ab^	0.26 ± 0.10 ^a^
P.a.35IB	0.13 ± 0.05 ^ab^	0.09 ± 0.05 ^c^	−0.01 ± 0.09 ^d^	0.13 ± 0.00 ^ab^	0.20 ± 0.07 ^a^	0.38 ± 0.10 ^a^
W.c.17MD	0.03 ± 0.04 ^abc^	1.10 ± 0.04 ^ab^	4.09 ± 0.05 ^a^	0.06 ± 0.07 ^bcd^	0.20 ± 0.08 ^a^	0.30 ± 0.12 ^a^
W.c.13ID	0.01 ± 0.11 ^abc^	1.09 ± 0.06 ^ab^	4.49 ± 0.26 ^a^	−0.06 ± 0.11 ^e^	0.20 ± 0.11 ^a^	0.26 ± 0.05 ^a^
P.a.10BE	0.02 ± 0.02 ^abc^	0.01 ± 0.03 ^cd^	0.04 ± 0.09 ^d^	0.06 ± 0.09 ^bcd^	0.21 ± 0.05 ^a^	0.30 ± 0.04 ^a^
P.a.16BB	−0.08 ± 0.04 ^c^	−0.09 ± 0.02 ^e^	0.10 ± 0.07 ^d^	0.00 ± 0.03 ^cde^	0.21 ± 0.07 ^a^	0.30 ± 0.07 ^a^
Control	0.00 ± 0.00 ^abc^	0.00 ± 0.00 ^de^	0.00 ± 0.00 ^d^	0.00 ± 0.00 ^de^	0.00 ± 0.00 ^b^	0.00 ± 0.00 ^b^

Results are expressed as means ± SD (*n* = 3). Means in the same column with different superscripts are significantly different (*p* < 0.05).

## Data Availability

Data is contained within the article.
